# Microwave-Assisted Degradation of Unsaturated Polyester Resin into HDPE-Modified Aerogels for Oil Adsorption and Oil/Water Separation

**DOI:** 10.3390/gels12100922

**Published:** 2026-10-14

**Authors:** Shixue He, Qianqian Qian, Daning Lang, Ronglan Wu

**Affiliations:** 1 Key Laboratory of Oil & Gas Fine Chemicals, Ministry of Education, Xinjiang Uygur Autonomous Region, School of Chemical Engineering, Xinjiang University, Urumqi 830017, China; 2 School of Mechanical Engineering & Center for Post-Doctoral Studies of Mechanical Engineering, Xinjiang University, Urumqi 830017, China

**Keywords:** unsaturated polyester resin, microwave-assisted depolymerization, UPR-derived products, HDPE-modified aerogel, oil adsorption, oil/water separation

## Abstract

Thermosetting unsaturated polyester resin (UPR) is difficult to reprocess because curing generates an irreversible three-dimensional network. Here, cured UPR was treated in a microwave-assisted hydrazine hydrate/NaOH medium at 70 °C for 30 min to produce a water-swellable UPR-derived product (NUPR). NUPR was combined with glutaraldehyde, subjected to freeze–thaw processing and freeze-drying, and subsequently modified with high-density polyethylene (HDPE) to fabricate porous aerogels. FTIR results were consistent with partial cleavage of ester-containing structures, while TGA and SEM revealed changes in thermal behavior and morphology. Among the tested aerogels, AAS-6 exhibited a density of approximately 0.036 g cm^−3^ and a porosity of 96.6%. After HDPE treatment, the aerogel exhibited a water contact angle of 135.2° while maintaining rapid oil wetting. Its 5 min liquid uptake for the tested oils and organic solvents ranged from 20.14 to 38.31 g g^−1^. After ten adsorption–regeneration cycles, 89–96% of the initial oil uptake was retained, depending on the oil, whereas uptake of the tested volatile solvents remained comparatively unchanged under the respective regeneration conditions. H-6 also enabled free-oil uptake from water and gravity-driven treatment of surfactant-stabilized water-in-oil (W/O) emulsions, yielding high separation efficiencies and markedly reduced dispersed-droplet signals after filtration. These results demonstrate a route for coupling the chemical conversion of cured UPR with fabrication of porous materials for oil uptake and W/O emulsion separation.

## 1. Introduction

Unsaturated polyester resin (UPR) is widely used in construction, transportation equipment, composite materials, and industrial products because of its high processing efficiency, favorable mechanical properties, and weather resistance [1,2]. Once cured, UPR forms an irreversible three-dimensional crosslinked network and cannot be reprocessed by conventional melt reshaping [3]. Chemical degradation provides a promising route for converting this robust network into reusable oligomeric or molecular products [4]. Therefore, developing efficient degradation and functional reutilization strategies for cured UPR is of considerable importance.

Current routes for processing cured UPR mainly include mechanical treatment [5], thermochemical conversion, and chemical degradation [6]. Mechanical treatment generally converts pulverized UPR into low-value fillers [7], whereas pyrolysis requires high temperatures and produces complex mixtures of gaseous, liquid, and carbonaceous products [8]. Chemical degradation can cleave labile bonds, such as ester linkages, and transform the crosslinked network into reusable oligomers or small molecules, making it more attractive for the preparation of functional feedstocks [9]. However, conventional hydrolysis, alcoholysis, and aminolysis often require high temperatures or long reaction times [10], and milder and faster intensification strategies remain desirable [11].

Microwave heating enables polar media to heat rapidly in an alternating electromagnetic field, thereby shortening heat-transfer pathways and increasing the overall heating rate [12]. Microwave-assisted processes have been applied to the chemical conversion of polyester-based materials and have shown potential for shortening reaction times and reducing processing temperatures [13]. Beyond the degradation step itself, directly converting UPR-derived products into functional materials provides an alternative route for increasing the utilization value of cured thermosetting wastes [14,15]. In this context, integrating mild UPR conversion with porous-material fabrication and subsequent surface-wettability regulation remains of particular interest.

Oil phases and organic solvents in industrial wastewater, oil spills, and emulsified effluents can cause persistent aquatic contamination [16,17]. Hydrophobic and oleophilic aerogels have attracted considerable attention for oil adsorption and oil/water separation because of their low density, high porosity, and continuous mass-transfer pathways [18,19]. Using UPR-derived degradation products as aerogel precursors may integrate resin conversion with the fabrication of functional separation materials, thereby increasing the utilization value of the polymer feedstock [20,21]. An et al. hydrolyzed the ester bonds of UPR at 80 °C for 5 h in a binary alkaline system consisting of diethylenetriamine and sodium hydroxide, and obtained a gel material capable of adsorbing dyes and heavy metal ions [22]. Wang et al. cleaved the ester groups of UPR at 140 °C in a potassium carbonate/ethylene glycol system to produce a styrene–maleic acid copolymer [23]. The resultant copolymer was physically crosslinked with polyvinyl alcohol to fabricate amphiphilic aerogels.

Here, microwave heating was employed to conduct the hydrazine hydrate/NaOH treatment of cured UPR at 70 °C for 30 min. The molar ratio of the two components was regulated to obtain water-swellable NUPR suitable for subsequent material fabrication. Rather than isolating defined degradation products, the recovered NUPR was directly used as a precursor for porous aerogel preparation through GA-assisted network formation, repeated freeze–thaw treatment, and freeze-drying. The resulting aerogel was subsequently treated with waste HDPE to regulate its surface wettability. Thus, the present work integrates UPR conversion, direct porous-material fabrication, and waste-HDPE-assisted wettability regulation within a single functional reutilization route.

## 2. Results and Discussion

**Figure 1:**
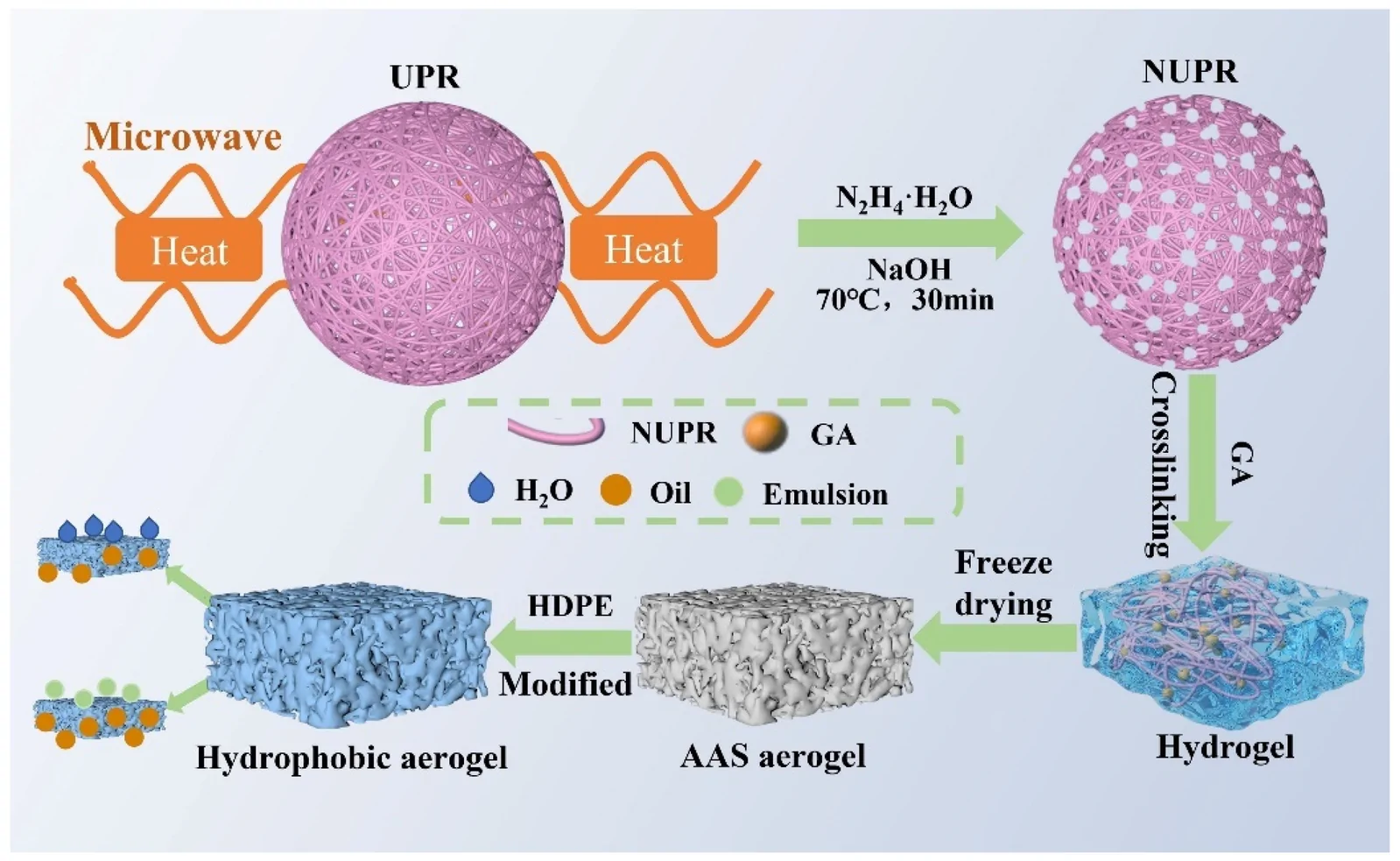
Schematic illustration of the preparation of NUPR, AAS aerogels, and HDPE-modified H-series aerogels.

The overall preparation process of NUPR, AAS aerogels, and HDPE-modified H-series aerogels is shown in Figure 1. First, cured UPR was pulverized and treated with hydrazine hydrate/NaOH under microwave heating at 70 °C for 30 min to obtain water-swellable NUPR through partial disruption of the ester-containing crosslinked structure. NUPR-3, which exhibited a favorable balance between swelling capacity and recovery yield, was selected for subsequent aerogel fabrication. NUPR-3 was then dispersed in deionized water, and glutaraldehyde was added as a crosslinking/network-forming component to facilitate hydrogel formation. After repeated freeze–thaw treatment and freeze-drying, porous AAS aerogels were obtained. Finally, the aerogels were treated with waste HDPE to regulate surface wettability, yielding the H-series aerogels.

### 2.1. Effect of the Hydrazine Hydrate/NaOH Ratio on the Structure and Properties of NUPR

**Figure 2:**
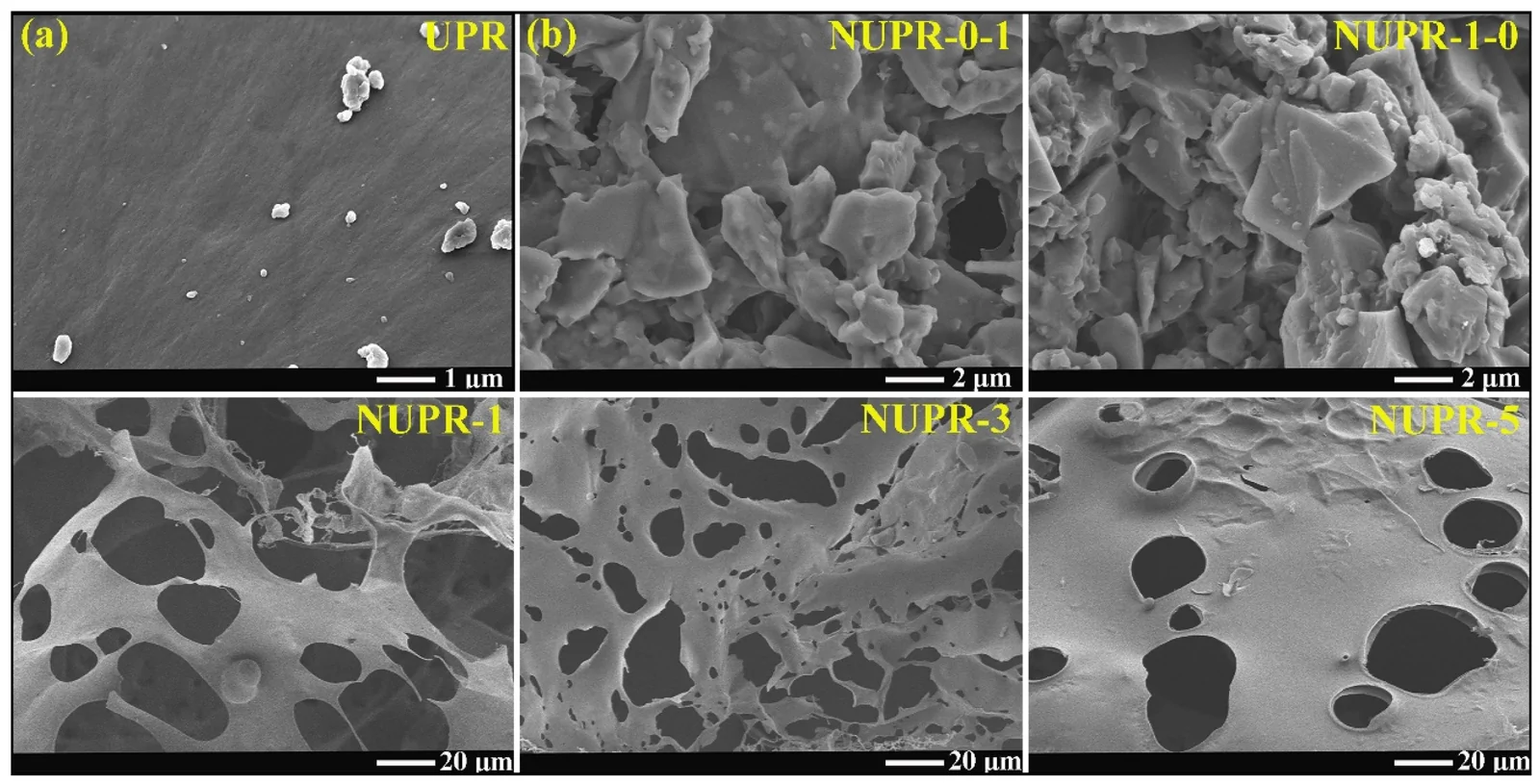
SEM images of pristine (**a**) UPR and (**b**) NUPR obtained using different reaction systems.

The surface of the pristine UPR was relatively smooth and compact, with no obvious cracks or pores (Figure 2a). After treatment with NaOH or hydrazine hydrate alone, NUPR-0-1 and NUPR-1-0 exhibited flake-like erosion, fragmented particles, and sparse porous regions. More pronounced morphological changes were observed for NUPR-1, NUPR-3, and NUPR-5 obtained using the combined hydrazine hydrate/NaOH system, which exhibited more developed porous regions (Figure 2b). Under the same magnification, NUPR-3 and NUPR-5 exhibited more developed porous morphologies than the samples obtained using hydrazine hydrate or NaOH alone. With increasing hydrazine hydrate content, the morphological changes became more pronounced. These observations indicate greater disruption of the original UPR crosslinked network under the tested conditions [24]. Together with the FTIR results, the morphological changes are consistent with partial cleavage of ester-containing structures and the formation or enrichment of polar functionalities.

**Figure 3:**
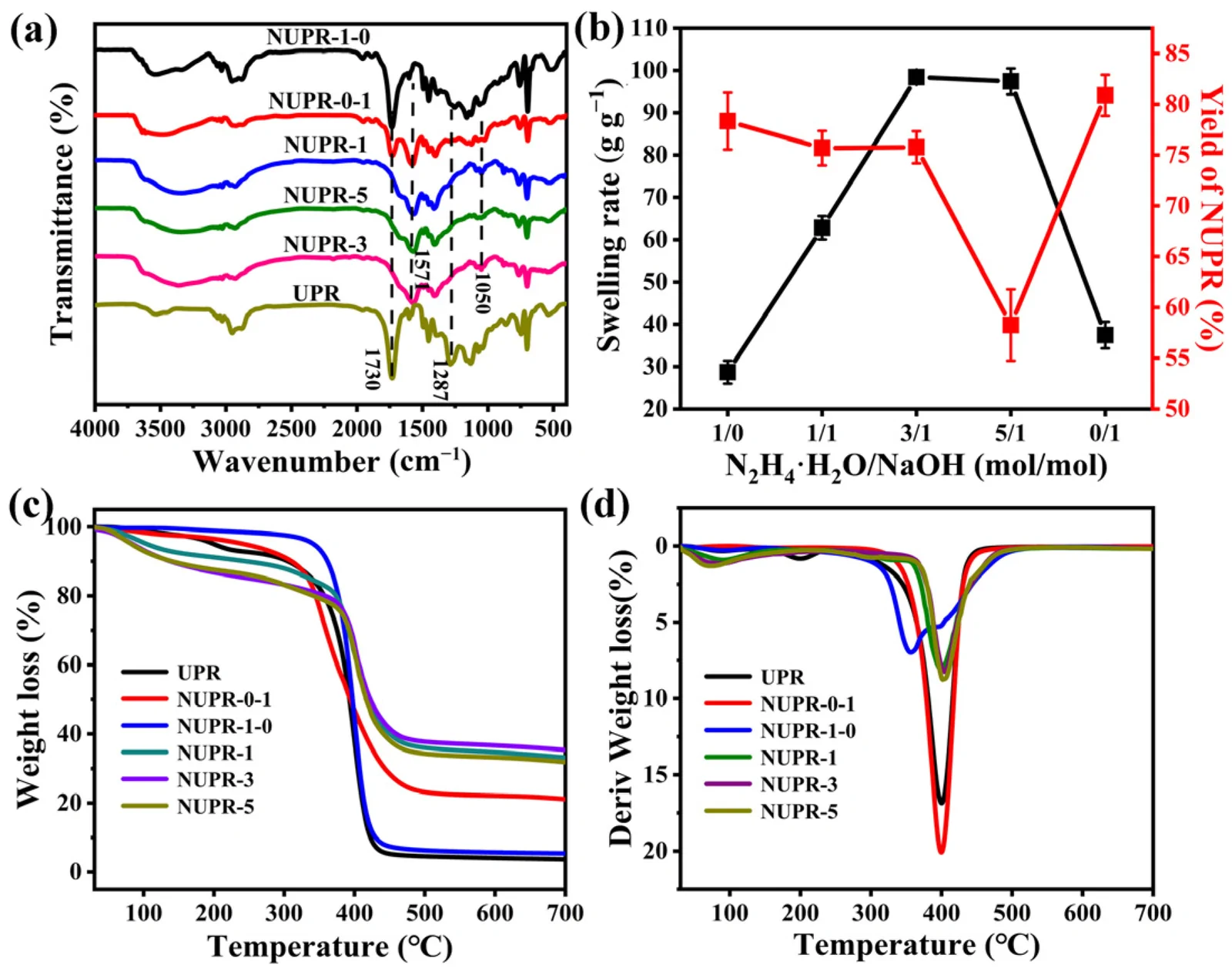
(**a**) FTIR spectra, (**b**) swelling ratios and yields, (**c**) TGA curves, and (**d**) DTG curves of UPR and NUPR.

Figure 3a shows the FTIR spectra of pristine UPR and the different NUPR samples. Compared with UPR, NUPR-1-0 prepared using hydrazine hydrate alone showed weakened ester-related bands near 1730 and 1287 cm^−1^, together with the appearance of a hydroxyl-related band at approximately 3537 cm^−1^. When NaOH was used alone, the ester bands were further weakened and the broad hydroxyl band became more pronounced. In the combined hydrazine hydrate/NaOH system, the bands near 1730 and 1287 cm^−1^ decreased markedly. With increasing hydrazine hydrate/NaOH molar ratio, the absorptions near 1571 and 1410 cm^−1^, assigned to carboxylate groups, and the broad hydroxyl-related band near 3385 cm^−1^ became progressively more pronounced. These spectral changes are consistent with partial cleavage of ester-containing structures and the formation or enrichment of additional polar functionalities. The bands near 3028, 1492, and 1451 cm^−1^, associated with the aromatic framework C-H and C=C vibration, remained detectable, suggesting that the aromatic-framework-related bands remained detectable after treatment.

From a chemical perspective, alkaline hydrolysis/saponification of ester linkages by NaOH may generate carboxylate- and hydroxyl-containing species, whereas hydrazine hydrate may also participate in nucleophilic reactions with ester-containing structures. Therefore, the formation of additional polar functionalities and the more open morphology may contribute to the enhanced water-swelling behavior of the NUPR products, thereby facilitating their subsequent use as precursors for porous aerogel fabrication.

The yields and swelling ratios of the NUPR samples are shown in Figure 3b. When hydrazine hydrate or NaOH was used alone (molar ratio 1:0, 0:1), the swelling ratio was below 40 g g^−1^. At a molar ratio of 1:1, the swelling ratio increased to approximately 60 g g^−1^, while ratios of 3:1 and 5:1 produced values approaching 100 g g^−1^. Meanwhile, the recovered solid yields of the 1:0, 0:1, 1:1, and 3:1 systems remained at approximately 80% or above, whereas the yield of the 5:1 system decreased to approximately 40%. The lower recovered yield at the highest hydrazine hydrate content may reflect more extensive disruption of the crosslinked structure and increased loss of soluble or dispersible fractions during washing. Considering both the high swelling ratio and the higher recovered yield relative to the 5:1 formulation, NUPR-3 prepared at a hydrazine hydrate/NaOH molar ratio of 3:1 was selected as the precursor for subsequent aerogel fabrication.

As shown in Figure 3c,d, pristine UPR and NUPR-1-0 underwent their main thermal decomposition within approximately 350–420 °C and exhibited low residual masses at 700 °C. In contrast, NUPR samples prepared in NaOH-containing systems showed higher residual masses at 700 °C, which may reflect changes in chemical composition during treatment, including contributions from carboxylate-containing structures and inorganic residues. The maximum mass-loss-rate temperature of NUPR-1-0 was approximately 350 °C, lower than the main decomposition peak at approximately 400 °C for pristine UPR and the NaOH-containing samples, indicating that hydrazine hydrate treatment alone had a more pronounced effect on thermal decomposition behavior. The slight mass loss below 100 °C was mainly attributed to adsorbed water and low-boiling residual species. Taken together, the FTIR and thermal-analysis results support structural changes in the ester-containing components after treatment, while the retained aromatic-related bands show that the aromatic framework remained detectable.

### 2.2. Structure and Physical Properties of AAS Aerogels

**Figure 4:**
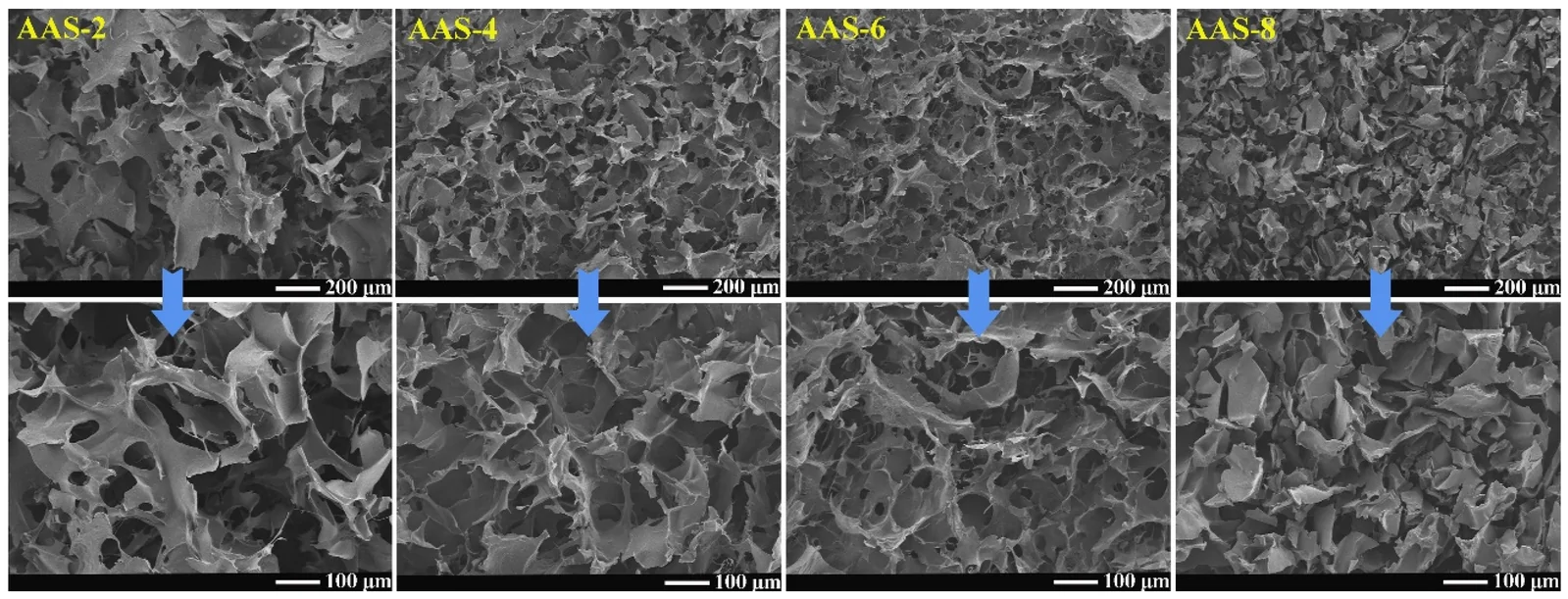
SEM images of AAS aerogels with different NUPR contents.

SEM images of the aerogels with different NUPR contents are shown in Figure 4. As the NUPR-3 content increased from 2 to 6 wt.%, the solid framework appeared progressively more continuous, accompanied by a more open porous morphology. When the NUPR content was further increased to 8 wt.%, locally denser regions were observed. These morphological differences indicate that the precursor concentration affects the structure retained after freeze-drying.

**Figure 5:**
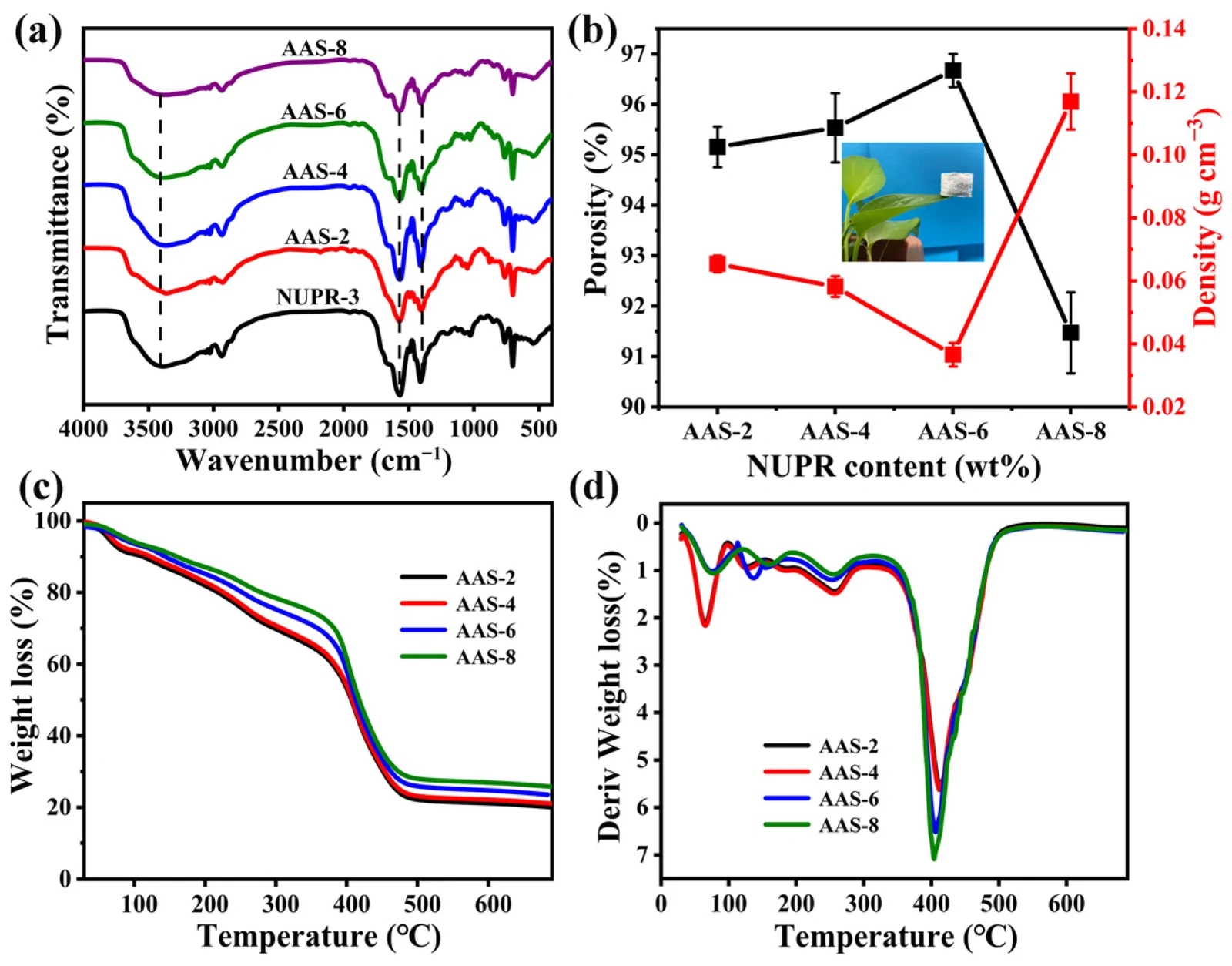
(**a**) FTIR spectra, (**b**) density and porosity of AAS aerogels (inset: photograph of the AAS-6 aerogel standing on the tip of a leaf), (**c**) TGA curves, and (**d**) DTG curves.

As shown in Figure 5a, the carboxylate-associated bands near 1571 and 1410 cm^−1^ and the aromatic-framework-related bands remained in AAS-2, AAS-4, AAS-6, and AAS-8. The broad band near 3385 cm^−1^ shifted to 3362, 3361, 3355, and 3353 cm^−1^, respectively, and gradually broadened with increasing NUPR content. The shift and progressive broadening of the OH-associated band indicate changes in the hydrogen-bonding environment and intermolecular interactions within the NUPR/GA network. Hydroxyl- and carboxylate-containing functionalities in NUPR may participate in these intermolecular interactions, while GA acts as a crosslinking/network-forming component during hydrogel formation.

As shown in Figure 5b, all AAS aerogels exhibited porosities above 91% and densities below 0.12 g cm^−3^, demonstrating the lightweight feature and high porosity of the aerogels. With increasing NUPR content, the density first decreased and then increased, whereas the porosity showed the opposite trend. Among the tested formulations, AAS-6 exhibited the lowest density of approximately 0.036 g cm^−3^ and the highest porosity of 96.6%, showing a favorable combination of low bulk density, high porosity, and a continuous framework under the present preparation conditions. The inset in Figure 5b shows that the AAS-6 aerogel can be supported on the tip of a leaf, consistent with its measured low bulk density.

As shown in Figure 5c,d, the AAS aerogels exhibited gradual mass loss below 300 °C, mainly because of the evaporation of adsorbed water and small amounts of low-molecular-weight species. The minor mass-loss-rate peaks between 200 and 300 °C may be associated with the initial decomposition of side groups, oligomeric segments, and crosslinked structures. A major mass-loss-rate peak appeared near 400 °C and corresponded to rapid decomposition of the NUPR-derived polymer framework. The four AAS samples showed similar main decomposition regions, suggesting that variation in precursor concentration did not substantially alter the principal thermal decomposition interval.

### 2.3. Wettability, Liquid Uptake, and Cyclic Performance

**Figure 6:**
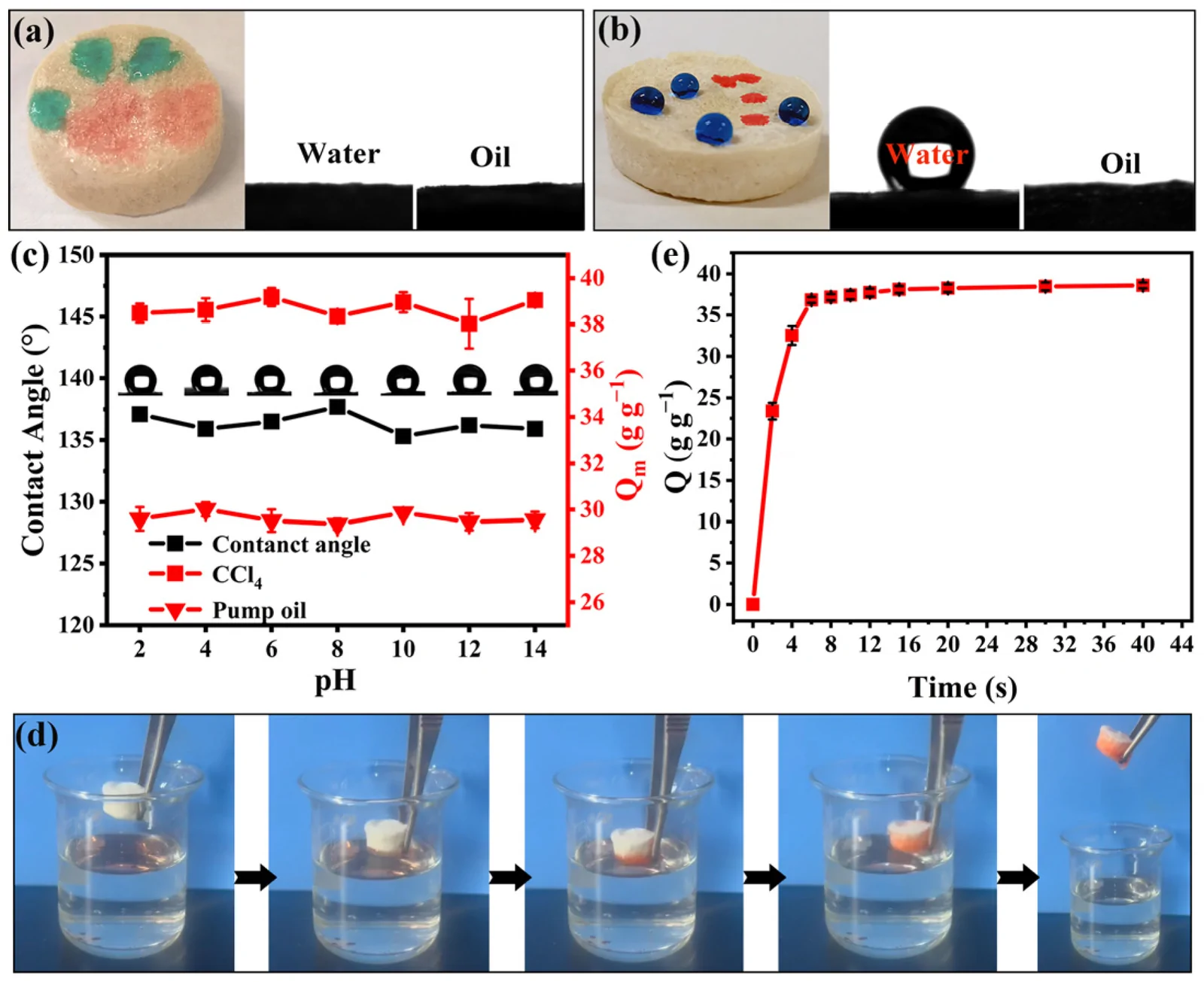
Wettability of (**a**) unmodified AAS and (**b**) HDPE-modified H-6; (**c**) contact angles of aqueous droplets with different pH values and 5 min liquid uptake capacities of H-6 for pump oil and carbon tetrachloride after 24 h immersion in the corresponding pH solutions; (**d**) selective uptake of floating oil from water; (**e**) time-dependent uptake of H-6 toward CCl_4_.

The unmodified AAS aerogels rapidly absorbed both water and oil droplets, and their water and oil contact angles were close to 0° (Figure 6a), indicating simultaneous hydrophilicity and oleophilicity. After HDPE treatment, the H-series aerogels retained rapid oil penetration into the pores, whereas water droplets remained approximately spherical on the surface (Figure 6b). H-6 exhibited a water contact angle of 135.2°. These results directly demonstrate that HDPE treatment substantially altered the surface wettability of the aerogel, imparting pronounced hydrophobicity while preserving its oleophilic character.

As shown in Figure 6c, when aqueous droplets with pH values ranging from 2 to 14 were directly placed on the dry H-6 surface, the measured contact angles remained above 135°, indicating pronounced water repellency toward both acidic and alkaline aqueous droplets. In addition, after H-6 was immersed in aqueous solutions with different pH values for 24 h, its 5 min liquid uptake capacities for pump oil and carbon tetrachloride remained at approximately 29 and 38 g g^−1^, respectively. These results indicate that H-6 maintained its hydrophobic wettability and liquid-uptake performance over a wide pH range under the tested conditions. Figure 6d further shows that H-6 rapidly removed dyed oil floating on water (Video S1), demonstrating selective uptake of the floating oil phase under the tested conditions. The time-dependent uptake of H-6 toward carbon tetrachloride is shown in Figure 6e. The uptake increased rapidly during the initial stage, reaching approximately 37 g g^−1^ within about 6 s, and subsequently approached a plateau of approximately 38 g g^−1^. The limited change thereafter indicates that carbon tetrachloride uptake had entered a relatively stable range within a short period.

**Figure 7:**
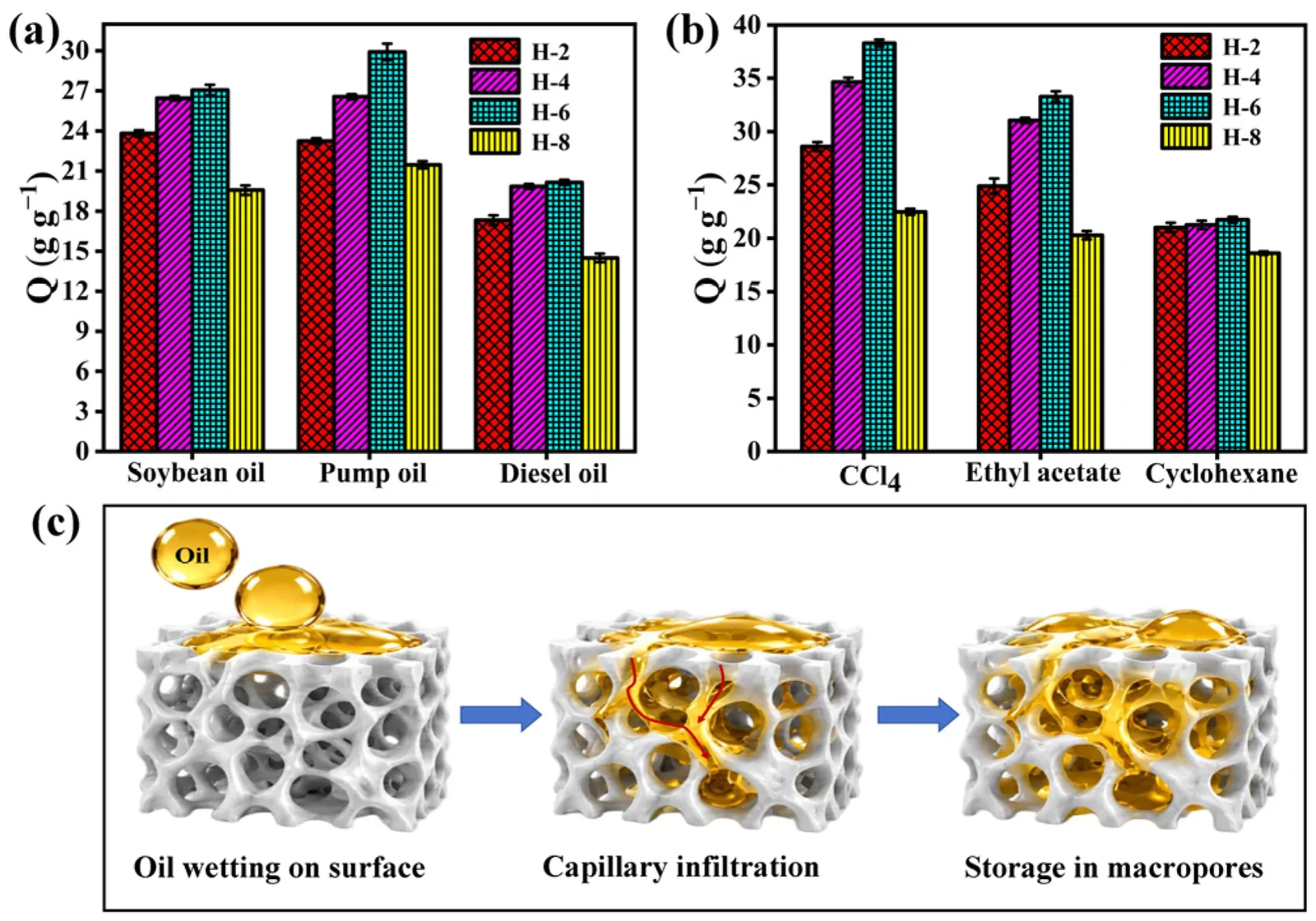
Liquid uptake capacities of H-2, H-4, H-6, and H-8 for (**a**) oils and (**b**) organic solvents; (**c**) schematic illustration of the proposed oil-uptake mechanism.

Figure 7a,b show the 5 min liquid uptake capacities of H-2, H-4, H-6, and H-8 for the tested oils and organic solvents. The liquid uptake first increased and then decreased with increasing NUPR-3 content, and H-6 exhibited the highest measured uptake among the tested formulations under the same experimental conditions. H-6 exhibited adsorption capacities of 20.14–38.31 g g^−1^, with the highest value obtained for carbon tetrachloride and the highest oil adsorption observed for pump oil. The differences among the tested liquids were related to their density, viscosity, surface tension, and retention within the pore network [25]. Gravimetric liquid uptake is governed by the combined effects of the aerogel structure and liquid properties. The low bulk density and high porosity of the AAS-6-derived framework provide a relatively large accessible void volume per unit mass for liquid accommodation. For a given accessible pore volume, liquid density affects the retained mass, whereas viscosity influences the infiltration and redistribution rate within the porous network. Surface tension and the oleophilic wettability of the HDPE-treated surface further affect capillary penetration and liquid retention. Consequently, the measured uptake reflects the combined effects of aerogel bulk density, accessible pore volume, liquid density, viscosity, surface tension, and interfacial wettability. Similar structure–interface coupling has been reported for hierarchical functional materials, in which architecture and interfacial chemistry jointly regulate transport and functional performance [26].

As illustrated in Figure 7c, the HDPE-treated surface promotes rapid wetting by oils and organic liquids, while the porous AAS-6-derived framework provides pathways and internal space for capillary infiltration and liquid accommodation. Therefore, the liquid-uptake performance of H-6 can be interpreted as a coupled effect of favorable liquid–surface interactions and porous architecture. This interpretation is also consistent with previous studies showing that surface modification can substantially alter adsorbate–surface interactions [27].

**Figure 8:**
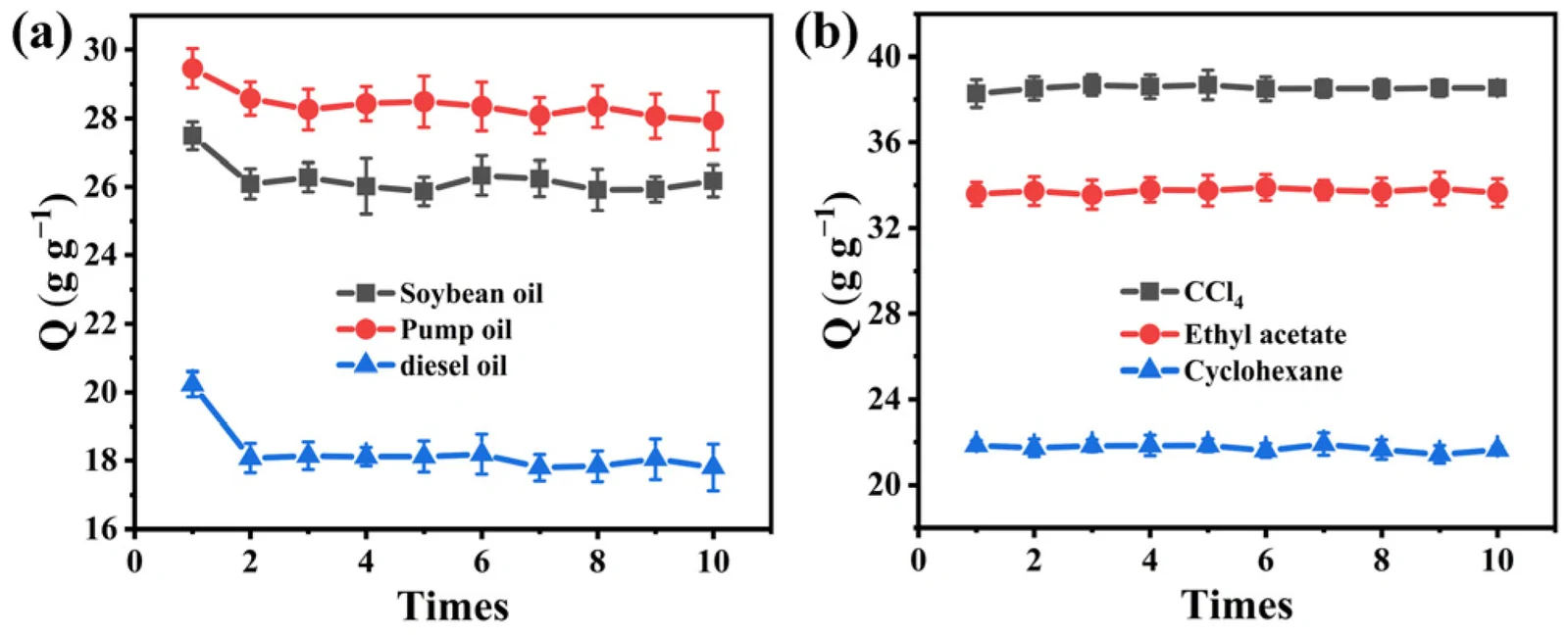
Cyclic liquid uptake performance of H-6 for (**a**) oils and (**b**) organic solvents.

H-6 was further evaluated over 10 adsorption–regeneration cycles (Figure 8a,b). Different regeneration methods were adopted according to the characteristics of the absorbed liquids: nonvolatile oils were removed by mechanical squeezing, whereas volatile organic solvents were removed by evaporation. For soybean oil, pump oil, and diesel, the liquid uptake decreased after the first regeneration cycle and then showed only minor variations during the subsequent cycles. After 10 cycles, approximately 89–96% of the initial oil uptake was retained, depending on the oil. The decrease in oil uptake may be associated with incomplete removal and residual retention of the relatively viscous oils within the porous structure during mechanical squeezing. For carbon tetrachloride, ethyl acetate, and cyclohexane, the liquid uptake changed only slightly over 10 cycles under the corresponding regeneration procedure. These results indicate that H-6 maintained relatively reproducible liquid-uptake performance over ten adsorption–regeneration cycles under the tested regeneration conditions.

As summarized in Table 1, H-6 exhibited a liquid uptake of 20.14–38.31 g g^−1^, which is within the relatively high range of the reported aerogel absorbents. Although some reported materials show higher maximum uptake values, H-6 retained approximately 89–96% of its initial oil uptake after 10 adsorption–regeneration cycles, which is higher than several of the comparison materials and comparable to the systems exhibiting high cyclic retention. Therefore, the main advantage of H-6 lies not in a single record-high uptake value, but in the favorable combination of relatively high liquid uptake and good cyclic retention. In addition, H-6 is derived from waste UPR and further treated using waste HDPE, providing a functional reutilization route for two waste-polymer streams.

### 2.4. Separation Performance for W/O Emulsions

**Figure 9:**
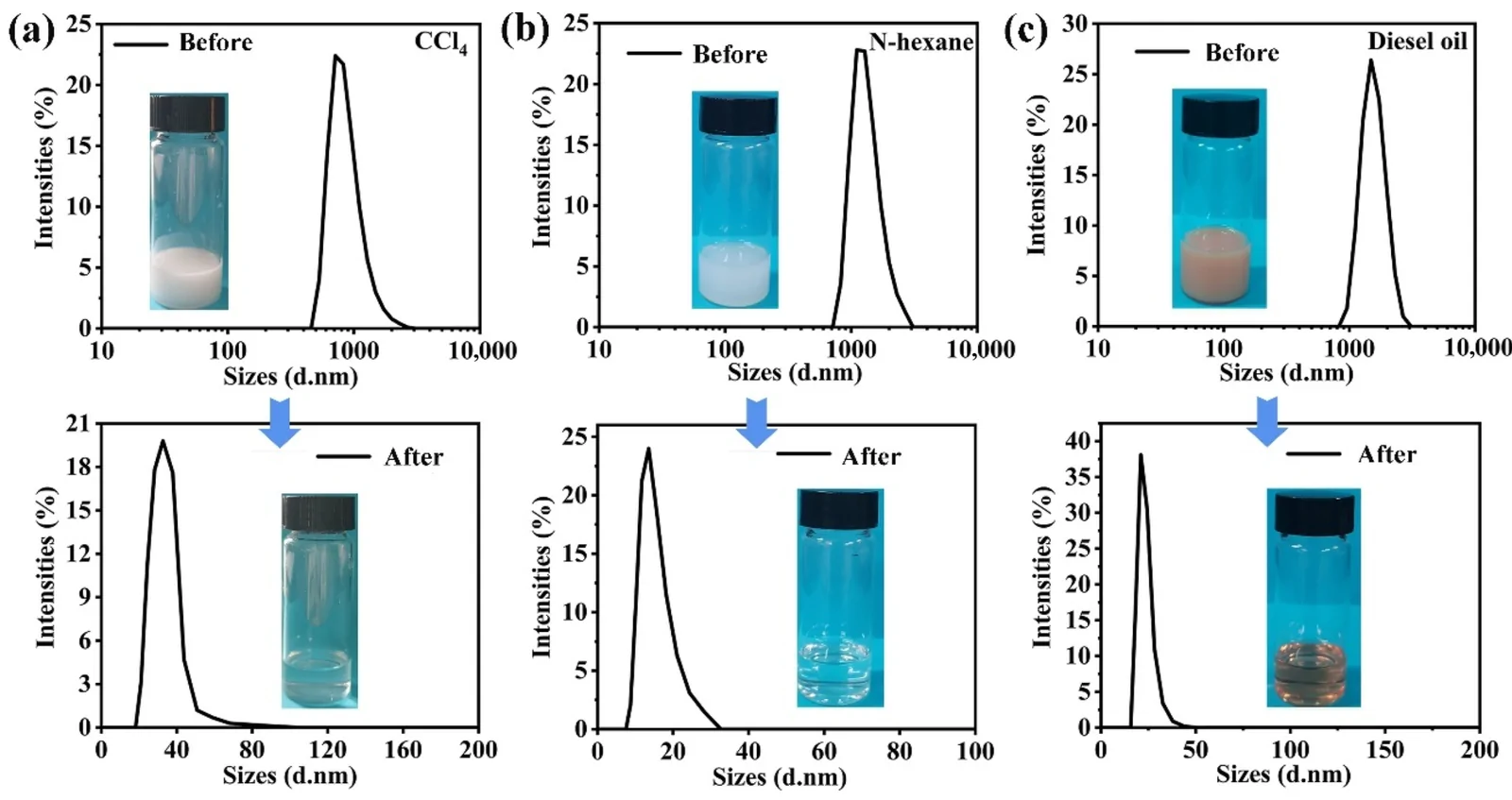
Optical images and apparent particle-size distributions of W/O emulsions before and after separation by H-6: (**a**) water/carbon tetrachloride, (**b**) water/n-hexane, and (**c**) water/diesel emulsions.

To evaluate the separation of surfactant-stabilized W/O emulsions by H-6, water/carbon tetrachloride, water/n-hexane, and water/diesel emulsions were prepared. Before separation, all three emulsions appeared milky. After gravity-driven filtration through H-6, the filtrates became visibly transparent (Figure 9). The main apparent particle-size distributions of the feed emulsions were approximately 650–1200, 960–1700, and 1200–2000 nm, respectively, whereas the corresponding apparent particle-size signals after separation decreased to approximately 25–50, 10–22, and 18–38 nm.

**Figure 10:**
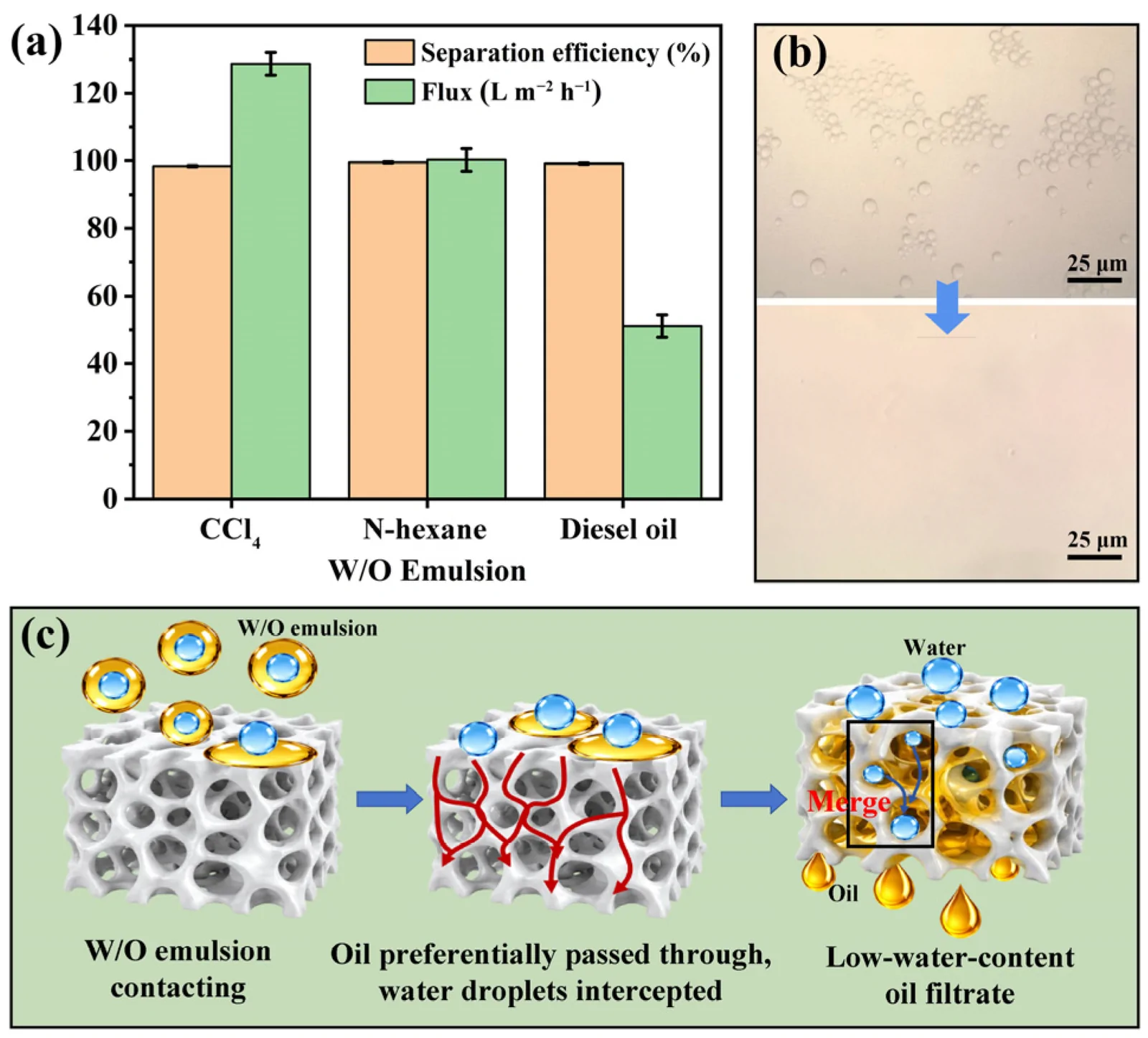
(**a**) Separation efficiency and permeation flux for three W/O emulsions; (**b**) optical microscopy images of the water/CCl_4_ emulsion before and after separation; (**c**) schematic illustration of the proposed W/O emulsion separation mechanism.

The separation efficiency and permeation flux of the three W/O emulsions were further measured, and the results are presented in Figure 10a. H-6 exhibited high separation efficiencies for water/carbon tetrachloride, water/n-hexane and water/diesel emulsions. Clear differences in permeation flux were observed among the three systems. The water/carbon tetrachloride emulsion showed the highest flux, followed by the water/n-hexane emulsion, whereas the water/diesel emulsion showed the lowest flux. These differences may be related to the different viscosities and interfacial characteristics of the continuous oil phases. Optical microscopy was further used to examine the water/carbon tetrachloride emulsion before and after separation (Figure 10b). A large number of micron-sized dispersed water droplets were observed in the feed emulsion, whereas far fewer dispersed water droplets were observed after filtration through H-6. Together with the quantitative water-content measurements, the particle-size and microscopic observations support the effective removal of dispersed aqueous droplets by H-6.

The W/O emulsion-separation behavior of H-6 is governed by the combined effects of selective wettability and porous transport pathways. The continuous oil phase preferentially wets the HDPE-treated surface and enters the porous framework, whereas the dispersed aqueous droplets experience unfavorable interactions with the hydrophobic surface. As the emulsion passes through H-6, water droplets may therefore be intercepted at the external surface, pore entrances, and locally constricted transport pathways. Some retained droplets may further collide and coalesce during filtration. Meanwhile, the continuous oil phase passes through the porous framework under gravity (Figure 10c). This proposed mechanism is consistent with the marked reduction in apparent dispersed-droplet signals, the optical-microscopy observations, and the quantitative decrease in water content measured after filtration. Overall, the HDPE-treated surface mainly provides selective wettability, while the porous framework facilitates oil-phase transport and contributes to the interception of dispersed aqueous droplets.

## 3. Conclusions

Microwave-assisted treatment of cured UPR in a hydrazine hydrate/NaOH medium at 70 °C for 30 min generated a water-swellable UPR-derived product that could be converted into a porous aerogel through glutaraldehyde treatment, freeze–thaw processing, and freeze-drying. FTIR data support partial cleavage of ester-containing structures, while the retained aromatic bands indicate that the aromatic framework was not completely destroyed. The resulting AAS-6 aerogel exhibited a density of approximately 0.036 g cm^−3^ and a porosity of 96.6%. After HDPE treatment, H-6 exhibited a water contact angle of 135.2° while retaining rapid oil wetting. H-6 showed 5 min liquid uptake capacities of 20.14–38.31 g g^−1^ for the tested oils and organic solvents. After ten adsorption–regeneration cycles, oil uptake remained at 89–96% of the initial values, depending on the oil, while uptake of the tested volatile solvents changed only slightly under the reported regeneration conditions. H-6 further enabled free-oil uptake from water and gravity-driven treatment of W/O emulsions, with high separation efficiencies and reduced dispersed-droplet signals after filtration. Overall, these results demonstrate the feasibility of coupling cured UPR conversion with porous-material fabrication for oil uptake and W/O emulsion treatment under the tested laboratory conditions. Further studies are required to evaluate longer-term cycling, mechanical durability, continuous-flow operation, performance in realistic multicomponent wastewater or emulsion systems, possible residual-chemical release or leaching, and scale-up of the preparation and separation processes.

## 4. Materials and Methods

### 4.1. Materials

Type 191 UPR was supplied by Guangdong Longteng Chemical Co., Ltd. (Sihui, China). Hydrazine hydrate (80%) was purchased from Tianjin Zhiyuan Chemical Reagent Co., Ltd. (Tianjin, China). Glutaraldehyde (GA) was obtained from Tianjin Kermel Chemical Reagent Co., Ltd. (Tianjin, China), and decalin was purchased from Sigma-Aldrich, St. Louis, MO, USA. Sodium hydroxide (NaOH), anhydrous ethanol, Span 80, ethyl acetate, n-hexane, cyclohexane, and carbon tetrachloride were commercially available reagents. High-density polyethylene (HDPE) was obtained from commercial plastic bags, while diesel, pump oil, and soybean oil were commercially available oils. All chemicals were used as received unless otherwise stated.

### 4.2. Preparation of NUPR

Type 191 UPR, the accelerator, and the curing agent were mixed uniformly at a volume ratio of 100:0.8:1 until a transparent mixture was obtained. The mixture was poured into a plastic mold, degassed under vacuum, and cured at room temperature for 2 h to obtain cured UPR. The cured UPR was pulverized and sieved to 80–100 μm. Hydrazine hydrate and NaOH were combined in a 250 mL round-bottom flask under an ice bath according to the specified molar ratios, while their total molar amount was fixed at 1.0 moL. For the 1:0 formulation, 80% hydrazine hydrate was used without NaOH. For the 0:1 hydrazine hydrate/NaOH formulation, NaOH was dissolved in deionized water instead of hydrazine hydrate. For the mixed formulations, NaOH was slowly added to 80% hydrazine hydrate under ice-bath conditions until dissolution was complete. UPR particles (4 g) were then added and dispersed uniformly. The mixture was transferred to an XH-300UP microwave catalytic synthesis instrument (Beijing Xianghu Science and Technology Development Co., Ltd., Beijing, China) and reacted at 70 °C under atmospheric pressure for 30 min. After the reaction, the solid product was collected by centrifugation at 8000 rpm for 5 min, washed three times with ethanol to remove residual reaction medium, and dried at 60 °C. The resulting UPR-derived product was denoted NUPR. The molar ratios of hydrazine hydrate to NaOH were set to 1:0, 0:1, 1:1, 3:1, and 5:1, and the corresponding samples were denoted NUPR-1-0, NUPR-0-1, NUPR-1, NUPR-3, and NUPR-5, respectively.

### 4.3. Fabrication of AAS Aerogels

NUPR-3 was dispersed in deionized water under magnetic stirring at 60 °C to prepare homogeneous precursor solutions containing 2, 4, 6, or 8 wt.% NUPR. GA (0.2 wt.%) was added to each solution, and the mixtures were stirred at room temperature for 30 min to form hydrogels. The hydrogels were frozen at −18 °C for 2 h and then naturally thawed at room temperature. After six freeze–thaw cycles, the samples were freeze-dried at −60 °C for 48 h to obtain porous NUPR-based aerogels. The samples were denoted AAS-2, AAS-4, AAS-6, and AAS-8 according to the NUPR content.

### 4.4. HDPE Modification

Commercial HDPE bags were cut into approximately 1 cm × 1 cm pieces and dissolved in decalin at 115 °C to prepare a 3 wt.% HDPE solution. The AAS aerogels were immersed in the HDPE solution for 1 min and then cooled naturally to room temperature. The samples were subsequently immersed in ethanol for 24 h, and the ethanol was replaced with fresh ethanol every 6 h to remove residual decalin. Finally, the aerogels were dried at 60 °C for 4 h. The resulting HDPE-modified aerogels were denoted H-2, H-4, H-6, and H-8, respectively, according to the NUPR content in the precursor formulation.

### 4.5. Characterization

Fourier transform infrared (FTIR) spectra were recorded using a Bruker Vertex 70 spectrometer (Bruker, Karlsruhe, Germany) over the range of 400–4000 cm^−1^. Thermogravimetric analysis (TGA) was performed using a TA Instruments SDT Q600 analyzer (TA Instruments, New Castle, DE, USA) under a nitrogen atmosphere from 30 to 700 °C at a heating rate of 10 °C min^−1^. The microstructures of the samples were observed by scanning electron microscopy (SEM; Tescan Mira LMS, Tescan, Brno, Czech Republic). Surface wettability before and after HDPE modification was characterized using a contact-angle goniometer (JJ2000B, Zhongchen, Shanghai, China) with a water-droplet volume of 2 μL. The apparent particle-size distributions of the emulsions before and after separation were measured using a Malvern Nano ZS90 analyzer (Malvern Panalytical, Malvern, UK). The water contents of the feed emulsions and the corresponding filtrates were determined using a Karl Fischer coulometric micro-moisture analyzer (HD-WS2, Shandong Horde Electronic Technology Co., Ltd., Weifang, China). All quantitative measurements were performed in triplicate.

### 4.6. Yield and Swelling Behavior of NUPR

The initial mass of UPR was denoted by *m*_0_, and the mass of dried NUPR was denoted by *m*_1_. After NUPR was allowed to swell in water for 4 h, the swollen sample was collected by vacuum filtration, and its mass was recorded as *m*_s_. The NUPR yield (*Y*) and mass swelling ratio (*S*) were calculated using Equations (1) and (2), respectively:

(1) $$Y=\frac{m_{1}}{m_{0}}\times 100\%$$

(2) $$S=\frac{m_{s}-m_{1}}{m_{1}}$$

where *Y* is the NUPR yield (%) and *S* is the mass swelling ratio (g g^−1^). All quantitative measurements were performed in triplicate.

### 4.7. Density and Porosity of the Aerogels

The aerogels were prepared as cylindrical specimens, and their dry mass (*m*_0_), diameter (*D*), and height (*h*) were measured. Three independent specimens were tested for each formulation, and the average value was reported. The bulk density (*ρ*) was calculated using Equation (3):

(3) $$\rho =\frac{4m_{0}}{\pi D^{2}h}$$

The porosity was measured using the ethanol-displacement method. An aerogel with a dry mass of *m*_0_ was vacuum-impregnated with anhydrous ethanol until no visible bubbles remained. After excess ethanol was removed from the surface, the saturated mass was recorded as *m*_2_. The porosity (*P*) was calculated using Equation (4):

(4) $$P=\frac{m_{2}-m_{0}}{\rho_{e}V}\times 100\%$$

where *V* is the apparent volume of the aerogel and *ρ*_e_ is the density of anhydrous ethanol (0.789 g cm^−3^).

### 4.8. Adsorption Measurements and Cyclic Regeneration

At room temperature, the dried HDPE-modified aerogels were immersed for 5 min in oils (diesel, pump oil, and soybean oil) or organic solvents (carbon tetrachloride, ethyl acetate, and cyclohexane). The samples were then suspended for 10 s to remove excess surface liquid and weighed. Three parallel measurements were conducted for each sample. The adsorption capacity (*Q*) was calculated using Equation (5):

(5) $$Q=\frac{m_{t}-m_{0}}{m_{0}}$$

where *m*_0_ and *m*_t_ are the masses of the aerogel before and after adsorption, respectively.

For regeneration, the absorbed nonvolatile oils were removed by mechanical squeezing, whereas the volatile organic solvents were removed by evaporation. The regenerated aerogels were subsequently reused for the next uptake test, and ten consecutive adsorption–regeneration cycles were performed for each liquid.

For the pH-dependent liquid-uptake stability test, H-6 samples were immersed in aqueous solutions with pH values ranging from 2 to 14 for 24 h. After the pH treatment, the samples were subsequently used to evaluate their 5 min liquid uptake capacities toward pump oil and carbon tetrachloride according to the procedure described above. All measurements were performed in triplicate.

### 4.9. Separation of W/O Emulsions

The aqueous and oil phases were mixed at a mass ratio of 2:98, and Span 80 was added at 0.1 wt.% relative to the total mass of the oil/water mixture. Carbon tetrachloride, n-hexane, and diesel were used as the oil phases. The mixtures were first stirred at 1000 rpm for 10 min and then at 500 rpm for 3 h to obtain stable W/O emulsions. H-6 was fixed in the separation device, and the emulsion was introduced from the top and allowed to pass through the aerogel under gravity. The filtrate was collected from the bottom. The emulsion separation efficiency (%) and permeation flux (L m^−2^ h^−1^) of H-6 were calculated via Equations (6) and (7).

(6) $$\mathrm{Separation}\;\mathrm{efficiency}=\frac{C_{\mathrm{feed}}-C_{\mathrm{filtrate}}}{C_{\mathrm{feed}}}\times 100\%$$

(7) $$F=\frac{4V}{\pi d^{2}\Delta t}$$

where *C*_feed_ and *C*_filtrate_ are the water content of the feed and filtrate, respectively. *V*, *d*, and Δ*t* represent the filtrate volume (L), effective diameter of the separation area (m), and filtration time (h), respectively.

## Figures and Tables

**Table 1:** Comparison of the oil and organic-liquid adsorption performance of different aerogels.

Aerogel	Reported Adsorption Capacity (g g^−1^)	Cycles/Retention	Reference
Methylcellulose/polyimide aerogels	25.19–28.44	5/82%	[28]
L-sodium alginate/cellulose nanofibrils aerogel	16–34	10/92.4%	[29]
Silylated bamboo fungus-based aerogel	20–42	10/95%	[30]
T-sodium alginate/lignin_x_/rGO-MTMS	5.296–13.214	10/39.08%	[1]
Fe_3_O_4_@chitosan/itaconic acid	17.7–43.8	10/67%	[31]
Wood aerogel/polydimethylsiloxane	9–20	Not reported	[32]
Ultra-flexible aerogel	7–15	20/84.8%	[33]
H-6	20.14–38.31	10/89–96%	This work

## Data Availability

Data will be made available on request.

## Supplementary Materials

The following supporting information can be downloaded at https://www.mdpi.com/article/10.3390/gels12100922/s1, Video S1: Selective uptake of floating oil from water.

## References

[1] Jiang Y.H., Zhang Y.Q., Gao C., An Q.D., Xiao Z.Y., Zhai S.R. (2022). Superhydrophobic aerogel membrane with integrated functions of biopolymers for efficient oil/water separation. Sep. Purif. Technol..

[2] Wang Y., Yan J., Wang J., Zhang X., Wei L., Du Y., Yu B., Ye S. (2020). Superhydrophobic metal organic framework doped polycarbonate porous monolith for efficient selective removal oil from water. Chemosphere.

[3] Yao Y., Wang B., Zhao F., Hu Z., Huang Y. (2022). Degradable and recyclable unsaturated polyester resin based on the selective cleavage of a hindered c–o bond. ACS Appl. Polym. Mater..

[4] An W., Zhang Y., Li J., Zhang S., Shen C., Liu X., Su Z., Xu S., Wang Y.Z. (2024). Fragmentation effect of solvent in recovery of unsaturated polyester resin and its composites. Compos. Part B Eng..

[5] García D., Vegas I., Cacho I. (2014). Mechanical recycling of GFRP waste as short-fiber reinforcements in microconcrete. Constr. Build. Mater..

[6] Liu L.J., Wang X.L., Liu Y.Z., Yang J.Y., Gu Z.Y., Chang T. (2024). Catalytic degradation of a thermosetting unsaturated polyester via a green coupled acid catalytic system. Green Chem..

[7] Barba B.J.D., Seko N., Madrid J.F., Penaloza D.P. (2023). Modified abaca fiber prepared by radiation-induced graft polymerization as a reinforcement for unsaturated polyester resin composites. Polym. J..

[8] Tian F., Wang X.L., Yang Y., An W., Zhao X., Xu S., Wang Y.Z. (2020). Energy-efficient conversion of amine-cured epoxy resins into functional chemicals based on swelling-induced nanopores. ACS Sustain. Chem. Eng..

[9] Wang B., Wang X., Xu N., Shen Y., Lu F., Liu Y., Huang Y., Hu Z. (2021). Recycling of carbon fibers from unsaturated polyester composites via a hydrolysis-oxidation synergistic catalytic strategy. Compos. Sci. Technol..

[10] Liu J., Wang S., Peng Y., Zhu J., Zhao W., Liu X. (2021). Advances in sustainable thermosetting resins: From renewable feedstock to high performance and recyclability. Prog. Polym. Sci..

[11] Elvitigala K.C.M.L., Mubarok W., Sakai S. (2024). Hydrogels with ultrasound-treated hyaluronic acid regulate CD44-mediated angiogenic potential of human vascular endothelial cells in vitro. Biomolecules.

[12] Zhao Q., Li X., Tian Z., Ma H., Hou X., Wang Y., Wang Y. (2022). Controlling degradation and recycling of carbon fiber reinforced bismaleimide resin composites via selective cleavage of imide bonds. Compos. Part B Eng..

[13] Khalaf H.I., Hasan O.A. (2012). Effect of quaternary ammonium salt as a phase transfer catalyst for the microwave depolymerization of polyethylene terephthalate waste bottles. Chem. Eng. J..

[14] Ye W.J., Xi J.F., Sun Y., Meng L.C., Bian H.Y., Xiao H.N., Wu W.B. (2023). Superelastic chitin nanofibril/chitosan aerogel for effective circulating and continuous oil-water separation. Int. J. Biol. Macromol..

[15] Pang C., Xu X., Xiao Y., Li W., Yang L., Pan X., Fang C., Mao X. (2026). Fabrication of self-crosslinking bamboo fiber aerogels for oil-water separation. Int. J. Biol. Macromol..

[16] Diao S., Liu H., Chen S., Xu W., Yu A. (2019). Oil adsorption performance of graphene aerogels. J. Mater. Sci..

[17] Zhang Z., Zhang C., Liu G., Lang D., Wang L., Yang C., Fu J., Wu R., Wang W. (2025). Adsorption behavior of amine-modified cotton stalk extract on congo red in aqueous solution. React. Funct. Polym..

[18] Qin Z.H., Mou J.H., Chao C.Y.H., Chopra S.S., Daoud W., Leu S.Y., Ning Z., Tso C.Y., Chan C.K., Tang S. (2021). Biotechnology of plastic waste degradation, recycling, and valorization: Current advances and future perspectives. ChemSusChem.

[19] Liu Q., Wang B., Jiang H., Lu Q., Wang L., Yang Y., Cheng R., Gao Q., Yang L., Du G. (2024). Optimization of durable superhydrophobic wood with superstrong ultraviolet resistance and chemical stability. Chem. Eng. J..

[20] Wang X.L., An W.L., Yang Y., Hu Z.Y., Xu S., Liao W., Wang Y.Z. (2019). Porous gel materials from waste thermosetting unsaturated polyester for high-efficiency wastewater treatment. Chem. Eng. J..

[21] Wang X.L., An W.L., Tian F., Yang Y., Zhao X., Xu P.P., Xu S., Wang Y.Z. (2020). High-efficiency hydrolysis of thermosetting polyester resins into porous functional materials using low-boiling aqueous solvents. ACS Sustain. Chem. Eng..

[22] An W., Wang X.L., Yang Y., Xu H., Xu S., Wang Y.Z. (2019). Synergistic catalysis of binary alkalis for the recycling of unsaturated polyester under mild conditions. Green Chem..

[23] Wang X.L., An W.L., Tian F., Yang Y., Zhao X., Xu P.P., Xu S., Wang Y.Z. (2021). Recycling waste thermosetting unsaturated polyester resins into oligomers for preparing amphiphilic aerogels. Waste Manag..

[24] Wang X.L., An W.L., Tian F., Yang Y., Xu H.X., Wang D., Zhao X., Xu P.P., Xu S., Wang Y.Z. (2020). Fast microwave-assisted hydrolysis of unsaturated polyester resin into column packing for rapid purifying of dye wastewater. J. Hazard. Mater..

[25] Jiang H., Li X.L., Wang J.H., Xu Q.Y., Wang Y.J., Deng C. (2025). Three-dimensional multiscale superhydrophobic thermoplastic polyurethane/sio_2_ nanowire composite sponges for durable and efficient oil-water separation. Surf. Coat. Technol..

[26] Wang Y.Y., Jiang C.J., Le Y., Cheng B., Yu J.G. (2019). Hierarchical honeycomb-like Pt/NiFe-LDH/rGO nanocomposite with excellent formaldehyde decomposition activity. Chem. Eng. J..

[27] Li Q., Huang Q., Ruan Y.F. (2022). Evaluation of modified peanut shell in the removal of Cr(VI) from aqueous solution. Desalin. Water Treat..

[28] Zhuo L., Ma C., Xie F., Chen S., Lu Z. (2020). Methylcellulose strengthened polyimide aerogels with excellent oil/water separation performance. Cellulose.

[29] Yang J., Xia Y., Xu P., Chen B. (2018). Super-elastic and highly hydrophobic/superoleophilic sodium alginate/cellulose aerogel for oil/water separation. Cellulose.

[30] Yi L., Xia Y., Tan Z., Fang X., Zhao L., Wu H., Guo S. (2020). Design of tubelike aerogels with macropores from bamboo fungus for fast oil/water separation. J. Clean. Prod..

[31] Yin Z., Sun X., Bao M., Li Y. (2020). Construction of a hydrophobic magnetic aerogel based on chitosan for oil/water separation applications. Int. J. Biol. Macromol..

[32] Wang K., Liu X., Tan Y., Zhang W., Zhang S., Li J. (2019). Two-dimensional membrane and three-dimensional bulk aerogel materials via top-down wood nanotechnology for multibehavioral and reusable oil/water separation. Chem. Eng. J..

[33] Du C., Chen Y., He S., Ruan C., Liu X., He C., Jin X., Chen Q., Ma Y., Chen G. (2022). Insight into ultra-flexible & robust silica aerogels based on diene synthesis reaction: Preparation and oil/water separation. Appl. Surf. Sci..

